# Inter-Method Agreement of a Laboratory-Developed Qualitative CMV PCR Assay Across Multiple Non-Plasma Clinical Specimens

**DOI:** 10.3390/v18040417

**Published:** 2026-03-27

**Authors:** Murat Aral, Ayfer Bakır, Cemal Çiçek, Elif Tuğçe Güner, Didem Özkan, Gülşah Ceylan Yağız, Mehmet Morkoç, Muhammed Furkan Kürkçü, Yusuf Üstün, Harun Erdal, Şevki Çelen, Emine Bahar Kurt, Serap Akçalı Duru, Asuman Nur Karhan, Ferda Özbay Hoşnut, Ayşegül Tok, Nilgün Eroğlu, Ferit Kulalı, Nurcan Hanedan, Yasemin Erol, Ahmet Kürşad Güneş, Göknur Yapar Toros, Ebru Oruç

**Affiliations:** 1 Department of Medical Microbiology, Ankara Etlik City Hospital, Ankara 06170, Türkiye; dr.ayfer.bakir@gmail.com (A.B.); drcemal06@gmail.com (C.Ç.); eliftugce06md@gmail.com (E.T.G.); didem.yigitt@gmail.com (D.Ö.); ceylan218@outlook.com (G.C.Y.); mehmet.morkoc93@gmail.com (M.M.); furkankurkcu@gmail.com (M.F.K.); 2 Department of Gastroenterology, Ankara Etlik City Hospital, Ankara 06170, Türkiye; yusufus06@gmail.com (Y.Ü.); drharunerdal@gmail.com (H.E.); 3 Department of Perinatology, Ankara Etlik City Hospital, Ankara 06170, Türkiye; sevkicelen@yahoo.com; 4 Department of Chest Diseases, Ankara Etlik City Hospital, Ankara 06170, Türkiye; baharkurt@yahoo.com (E.B.K.); akcalis@hotmail.com (S.A.D.); 5 Department of Pediatric Gastroenterology and Hepatology, Ankara Etlik City Hospital, Ankara 06170, Türkiye; asunurkar83@gmail.com (A.N.K.); ferdaozbay72@yahoo.com (F.Ö.H.); ayse.cb@gmail.com (A.T.); 6 Department of Pediatric Hematology and Oncology, Ankara Etlik City Hospital, Ankara 06170, Türkiye; dr-nilguner@hotmail.com; 7 Department of Neonatology, Ankara Etlik City Hospital, Ankara 06170, Türkiye; fkulali@hotmail.com (F.K.); drhanedan@gmail.com (N.H.); 8 Department of Ophthalmology, Ankara Etlik City Hospital, Ankara 06170, Türkiye; yasemin_oz@yahoo.com; 9 Department of Hematology, Ankara Etlik City Hospital, Ankara 06170, Türkiye; ahmetkgunes@gmail.com; 10 Department of Infectious Diseases, Ankara Etlik City Hospital, Ankara 06170, Türkiye; goknuryapar06@hotmail.com (G.Y.T.); ebru.oruc_@hotmail.com (E.O.)

**Keywords:** cytomegalovirus, real-time PCR, qualitative PCR, non-plasma clinical samples, inter-method agreement

## Abstract

Background: This study evaluated the inter-method agreement of an in-house qualitative CMV real-time PCR assay for the detection of cytomegalovirus (CMV) DNA in various non-plasma clinical specimen types, in comparison with a commercially available comparator assay. Methods: In this prospective comparative study, 186 clinical specimens—including bronchoalveolar lavage fluid (BALF), stool, urine, colonoscopic biopsy, amniotic fluid, and intraocular fluid—were analyzed. A total of 166 samples with valid results from both test systems were included in the inter-method comparison. CMV DNA was detected using the in-house qualitative PCR assay in parallel with the comparator assay (artus^®^ CMV QS-RGQ kit). Agreement was assessed using positive percent agreement (PPA), negative percent agreement (NPA), overall percent agreement (OPA), and Cohen’s kappa coefficient (κ), in accordance with CLSI EP12-A2 recommendations. Results: Substantial overall inter-method agreement was observed when all specimens were evaluated collectively (κ = 0.66). Agreement metrics were highest in stool, urine, and invasive specimens, whereas BALF samples demonstrated comparatively lower agreement, reflecting potential matrix-related analytical variability. Conclusion: The laboratory-developed qualitative CMV PCR assay demonstrated substantial inter-method agreement with the comparator assay across multiple non-plasma specimen types. The findings highlight specimen-specific variability in qualitative CMV DNA detection and represent analytical concordance between two molecular assays rather than definitive clinical diagnostic accuracy or viral load quantification.

## 1. Introduction

Cytomegalovirus (CMV) is a DNA virus belonging to the *Betaherpesvirinae* subfamily of the *Herpesviridae* family and is widely prevalent worldwide. While CMV infection is mostly asymptomatic or mild in immunocompetent individuals, it can lead to serious clinical conditions in immunocompromised patients. Particularly, recipients of solid organ and hematopoietic stem cell transplants, individuals with inflammatory bowel disease, and newborns at risk for congenital infections are among the high-risk groups for CMV-related morbidity and mortality [1,2]. Early detection of CMV infection in these patient groups is critically important for the timely initiation of antiviral therapy and the prevention of clinical complications [2,3]. Therefore, the methods used in the laboratory diagnosis of CMV infection must demonstrate reliable analytical performance and clinical relevance.

Currently, serological methods, virological culture, and molecular techniques are used in the diagnosis of CMV. Polymerase chain reaction (PCR), a molecular method, is increasingly preferred in clinical practice due to its high analytical sensitivity and specificity, reproducibility, ability to detect CMV DNA even at low viral loads, and rapid turnaround time. These methods enable direct detection of the viral genome and, in quantitative formats, viral load monitoring. They are widely used in routine clinical laboratory practice because they offer the possibility of quantitative or qualitative assessment [4,5,6,7,8].

While plasma samples are commonly used for diagnosing CMV infections, the analysis of non-plasma clinical samples such as stool, urine, bronchoalveolar lavage fluid (BALF), and various tissue biopsies is critical for assessing the localization and extent of infection. Most commercially available real-time PCR kits are optimized for standardized plasma samples, and data regarding their analytical performance and inter-method agreement in different clinical specimen types remain limited [9,10,11,12,13,14]. However, it has been shown that in some cases, CMV DNA can be detected in tissue samples even when it is negative in serum or plasma samples, highlighting the diagnostic potential of non-plasma specimens [15].

However, the performance of CMV PCR tests can vary depending on multiple factors, such as the test platform used, the targeted viral genome region, the nucleic acid extraction method, and the type of clinical sample analyzed [5,16]. The presence of PCR inhibitors, low viral load levels, and heterogeneous distribution of CMV in tissue or body fluids, particularly in plasma-free clinical samples, can lead to inconsistent results between assays, underscoring the need for comparative analytical evaluation in these specimen types [16,17].

In the literature, studies comparing different commercial or laboratory-developed CMV PCR tests have frequently reported specimen-type-based differences, and inter-method agreement may vary depending on the sample matrix [18,19,20]. This situation highlights the importance of new CMV PCR assays undergoing comparative evaluation across different clinical sample types before broader laboratory implementation. The aim of this study was to comparatively evaluate a laboratory-developed qualitative CMV PCR assay against a commercially available comparator assay across different non-plasma clinical specimen types and to assess its performance characteristics under routine laboratory conditions. In this respect, the originality of the study lies not in introducing a novel PCR technology per se, but in providing a structured, specimen-type-based inter-method agreement evaluation across multiple specimen matrices. Because most commercial CMV PCR assays are primarily validated for plasma specimens, matrix-specific inter-method agreement data for non-plasma clinical samples remain limited. This study provides a structured, specimen-stratified agreement evaluation across diverse clinical matrices under routine laboratory conditions.

## 2. Materials and Methods

### 2.1. Study Design and Clinical Specimens

This study was a prospective comparative analytical study designed to evaluate the inter-method agreement of a qualitative real-time PCR assay for the detection of cytomegalovirus (CMV) DNA across different non-plasma clinical specimen types. The kit evaluated in the study was developed by PharmaLine (PharmaLine Health Services Ind. Trade Inc., Istanbul, Türkiye).

A total of 186 clinical samples collected between June 2025 and September 2025 were included in the study; however, 166 samples for which valid positive or negative results could be obtained with both test systems were included in the inter-method comparison analysis. All specimens included in the analysis were tested in parallel with both the laboratory-developed CMV PCR assay and the artus^®^ CMV QS-RGQ (QIAGEN, Hilden, Germany) comparator assay. A total of 20 samples were excluded from the analysis due to internal control failure or technical reasons. Samples showing internal control failure were excluded to ensure analytical validity and to avoid bias in inter-method agreement calculations, as such failures may indicate PCR inhibition or extraction inefficiency. Most excluded samples consisted of specimen types with a high inhibition potential, such as BALF and stool, and this distribution is presented in Supplementary Tables S1 and S2. This is consistent with known PCR inhibition in complex biological matrices.

The qualitative PCR kit evaluated in this study (PharmaLine Health Services Ind. Trade Inc., Istanbul, Türkiye) was developed in-house, and its performance was assessed through parallel testing and inter-method agreement analysis with a commercially available comparator assay. The primary objective of this study was to evaluate qualitative inter-method agreement between two PCR assays rather than to establish clinical diagnostic accuracy.

### 2.2. Clinical Setting and Sample Collection

The clinical samples included in this study were obtained from pediatric and adult patients referred from gastroenterology, pulmonology, ophthalmology, perinatology, hematology, pediatric gastroenterology and hepatology, neonatology, and pediatric hematology and oncology clinics of Ankara Etlik City Hospital with suspected CMV infection. All clinical specimens were obtained from patients in whom CMV infection was considered in the differential diagnosis based on clinical, laboratory, and/or radiological findings, as part of routine diagnostic workup. Suspected CMV infection was defined based on clinical manifestations such as persistent or unexplained fever, gastrointestinal symptoms (e.g., diarrhea, abdominal pain, gastrointestinal bleeding), respiratory symptoms with radiological findings suggestive of pneumonia, cytopenia, elevated inflammatory markers, or organ-specific findings (e.g., visual symptoms, fetal abnormalities), particularly in immunocompromised patients. All samples were collected by the relevant clinics as part of the routine clinical diagnostic process, in appropriate sterile containers, and delivered promptly to the Medical Microbiology Laboratory within a short period of time, in accordance with biosafety and pre-analytical quality criteria. Samples received in the laboratory were recorded, anonymized by concealing patient identification information, and stored frozen at −80 °C until analysis to preserve nucleic acid stability.

### 2.3. Inclusion and Exclusion Criteria

This study included clinical samples obtained from pediatric and adult patients referred from gastroenterology, pulmonology, ophthalmology, perinatology, and pediatric infectious diseases clinics for CMV infection testing and submitted to the Medical Microbiology Laboratory. The clinical specimens included BALF, stool, urine, colonoscopic biopsy, amniotic fluid, and intraocular fluid samples. Only samples that yielded valid positive or negative results with both the laboratory-developed CMV PCR assay and the comparator assay were included in the analysis.

Serum, plasma, and whole blood samples were not included in the study design. In addition, samples that failed the internal control during nucleic acid extraction or PCR, as well as samples that could not be evaluated by both test systems due to technical reasons, were excluded from the analysis.

### 2.4. Pre-Extraction Preparation of Clinical Samples

In this study, nucleic acid isolation was performed directly on urine and intraocular fluid samples without any pre-processing, using the volume recommended in the kit protocol. In contrast, tissue, stool, and BALF samples were pre-processed according to manufacturer protocols prior to extraction.

Stool samples were prepared with PharmaSEP buffer (Lot No: PHSP2024-50/PHSP2024-100; Pharmaline Health Services Ind. Trade Inc., Istanbul, Türkiye); 100 mg of solid samples and 100 µL of liquid samples were added to 900 µL of buffer. Samples were vortexed, subjected to freeze–thaw treatment, and the supernatant obtained after centrifugation was used for extraction.

BALF samples were treated with 5× dithiothreitol (DTT, Sigma-Aldrich, St. Louis, MO, USA) (DTT: sample = 1:4), and after incubation and homogenization, a volume of 300 µL was extracted. BALF samples were collected as part of the routine diagnostic evaluation upon clinicians’ request in patients with suspected CMV pneumonia or presumed pulmonary involvement in immunosuppressed individuals.

Tissue samples were prepared by homogenization according to the manufacturer’s instructions for the Molecision Nucleic Acid Extraction Kit (SNIBE Co., Ltd., Shenzhen, China) and included in the extraction process.

### 2.5. Nucleic Acid Extraction

Total nucleic acid isolation from clinical samples was performed using the Molecision Fully Automated Nucleic Acid Purification System (MP-96) device. Extraction procedures were performed according to the manufacturer’s instructions using the SNIBE Nucleic Acid Extraction Kit (Shenzhen New Industries Biomedical Engineering Co., Ltd., Shenzhen, Guangdong, China). The elution volume was standardized to 50 µL for all samples. The obtained nucleic acids were stored under appropriate conditions according to the manufacturer’s recommendations until PCR analysis. To minimize pre-analytical variability, nucleic acids were extracted once per specimen, and the same extract was aliquoted and tested in parallel with both PCR assays.

### 2.6. Real-Time PCR Design

The US17 gene, a conserved segment of the CMV genome, was targeted to detect CMV DNA. The human β-actin gene served as an internal control to assess extraction efficiency and PCR inhibition. Although the assay was qualitative in nature, the experimental design and reporting followed the relevant components of the MIQE guidelines [21].

Accordingly, primer and probe sequences were fully reported, an internal control was included to monitor extraction efficiency and PCR inhibition, and replicate testing was not performed, as the assay was qualitative.

Primer and Probe Design:

The primer and probe sequences used for the CMV target are as follows:

- CMV US17 forward primer: 5′-TCTCTGTACCTCCCGCAAAA-3′
- CMV US17 reverse primer: 5′-AGACAAACTCATCGCTTGGA-3′
- CMV US17 probe: 5′-FAM-TGACCTGGTTATCGTCACGCG-BHQ-3′
- Primer and probe sequences for the β-actin gene used as an internal control:
- β-actin forward primer: 5′-CACCATTGGCAATGAGCGGTT-3′
- β-actin reverse primer: 5′-TAGTTTCGTGGATGCCACAGG-3′
- β-actin probe: 5′-HEX-CACTCTTCCAGCCTTCCTTCCTGGG-BHQ-3′

PCR Components and Conditions:

PCR were prepared in a total volume of 20 µL. The reaction mixture consisted of the following components:

- A total of 10 µL RapidXFire™ qPCR Master Mix (2×)
- CMV primers: 500 nM (each)
- CMV probe: 125 nM
- β-actin primers: 250 nM (each)
- β-actin probe: 62.5 nM
- A total of 5 µL of template nucleic acid

In each study, a negative control (NTC) and a reaction blank containing only the reaction components were included for contamination control purposes.

Thermal Cycling Conditions:

The PCR amplification was carried out on a Bio-Rad CFX96 Touch Real-Time PCR Detection System (Bio-Rad Laboratories Inc., Hercules, CA, USA) under the following conditions: an initial denaturation step at 95 °C for 5 min, followed by 44 amplification cycles consisting of denaturation at 95 °C for 10 s and annealing/extension at 60 °C for 45 s.

The CMV PCR assay used in this study was designed as a qualitative test. Therefore, MIQE guideline criteria specific to quantitative analyses, such as the generation of standard curves, calculation of PCR efficiency, and absolute quantification, were not applied. However, all MIQE components relevant to qualitative PCR were fully implemented, including target gene definition, primer and probe sequences, amplification conditions, nucleic acid extraction method, use of an internal control, positive and negative controls, and clearly defined criteria for result interpretation.

Fluorescence signal measurement was performed at the binding/extension step of each cycle. Although quantitative analysis was not the aim of the study, it was conducted by considering the methodological criteria included in the MIQE guidelines, which can be adapted to qualitative real-time PCR applications [21].

US17 Region and In Silico Analysis:

To assess the conservation of the US17 target region, an in silico alignment analysis was performed on available CMV genomes, demonstrating the absence of significant mismatches within the primer and probe binding regions. This analysis is provided as Supplementary Material (Table S3).

### 2.7. Comparator Assay

The artus^®^ CMV QS-RGQ kit (QIAGEN, Hilden, Germany) was used as the comparator assay for inter-method agreement analysis. The comparator assay was not considered a clinical gold standard for all non-plasma specimen types. The commercially available comparator assay was performed in parallel with the laboratory-developed CMV PCR assay on the same extracted nucleic acid material, and results were classified qualitatively (positive/negative) in accordance with the manufacturer’s recommendations.

### 2.8. Sample Size

In this study, the sample size was determined based on the inclusion of all clinical samples available during the study period that yielded valid results with both test systems. A formal a priori power calculation was not performed because the study aimed to evaluate inter-method agreement using consecutively collected routine clinical specimens rather than to estimate clinical diagnostic accuracy.

### 2.9. Statistical Analysis

All statistical analyses were performed using IBM SPSS Statistics v26.0 software. In the absence of a clinical gold standard for non-plasma specimens, inter-method agreement between the two assays was assessed in accordance with CLSI EP12-A2 using positive percent agreement (PPA), negative percent agreement (NPA), overall percent agreement (OPA), and Cohen’s kappa coefficient.

Positive percent agreement (PPA), negative percent agreement (NPA), and overall percent agreement (OPA) were calculated from true positive (TP), false positive (FP), false negative (FN), and true negative (TN) results. Inter-method qualitative agreement was assessed using Cohen’s kappa coefficient (κ), and interpretation was based on the classification proposed by Altman [22].

Ninety-five percent confidence intervals were calculated using the Wilson score method. The calculation and reporting of agreement metrics were performed in accordance with the Clinical and Laboratory Standards Institute (CLSI) EP12-A2 guideline for qualitative diagnostic tests [23].

## 3. Results

### 3.1. Distribution of Clinical Specimens

A total of 166 non-plasma clinical specimens were included in the analysis. The distribution of specimen types and the overall CMV DNA positivity rates are presented in Table 1, with CMV positivity varying across different specimen types.

### 3.2. Agreement Between the Laboratory-Developed Qualitative CMV PCR Assay and the Comparator Assay by Specimen Type

**Figure 1:**
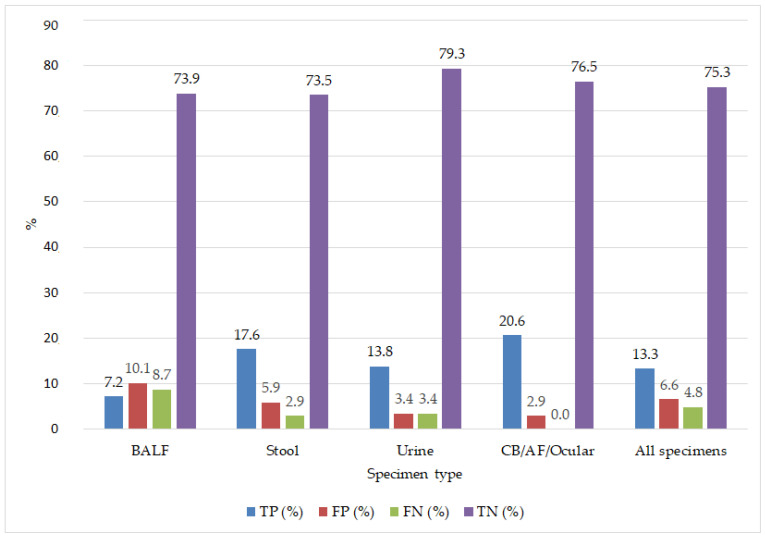
Agreement patterns between the laboratory-developed qualitative CMV PCR assay and the comparator assay across different specimen types.

The agreement between the laboratory-developed qualitative CMV PCR assay and the comparator assay was further evaluated according to specimen type. The percentage distribution of true-positive, true-negative, false-positive, and false-negative results by specimen type is illustrated in Figure 1.

The inter-method agreement metrics between the comparator assay and the laboratory-developed qualitative CMV PCR assay are presented in Table 2, stratified by specimen type.

In BALF samples, the PPA was 45.5%, the NPA was 87.9%, and the OPA was 81.2%. The Cohen’s kappa coefficient was 0.36, signifying a weak degree of concordance between the approaches. In stool samples, a strong concordance was observed with PPA at 85.7%, NPA at 92.6%, OPA at 91.2%, and Cohen’s κ at 0.79. In urine samples, the PPA was 80.0%, NPA at 95.8%, and OPA at 93.1%. The Cohen’s kappa coefficient was 0.74, indicating substantial agreement. In the group including colonoscopic biopsy, amniotic fluid, and intraocular fluid samples, the PPA was 100%, the NPA was 96.3%, and the OPA was 97.1%, indicating substantial concordance (Cohen’s κ = 0.93).

Upon evaluating all samples collectively, the overall concordance between the test and comparator assay was determined to be PPA 73.3%, NPA 91.9%, OPA 88.6%, and Cohen’s κ = 0.66, signifying a substantial degree of overall agreement.

### 3.3. Analytical Sensitivity

**Figure 2:**
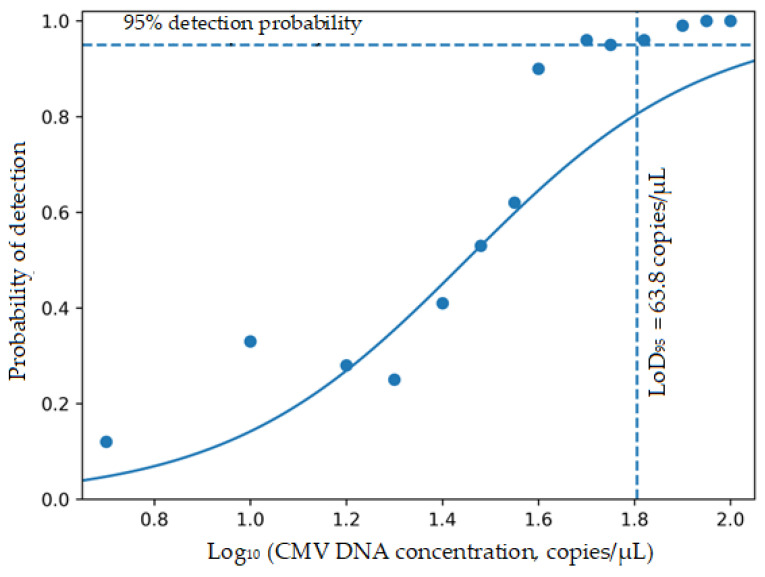
Probit dose–response curve for determining the LoD_95_. Detection probability (n_pos_/n_total_) obtained from replicate testing (n = 24 per concentration level) was plotted against log_10_-transformed CMV DNA concentration (copies/µL) and fitted using a probit regression model. The horizontal dashed line represents the 95% detection threshold, while the vertical dashed line indicates the corresponding LoD_95_ value (63.8 copies/µL).

The LoD_95_ for the CMV qPCR test, as determined by probit regression analysis using serial dilutions of plasmid DNA, was 63.8 copies/µL, corresponding to a 95% probability of detection (Table 3, Figure 2). Considering the extraction and elution volumes, this corresponds to approximately 1.6 × 10^4^ copies/mL of input material under the analytical conditions tested.

The relationship between target concentration and detection probability was evaluated using a probit regression model, and all model parameters were found to be statistically significant (*p* < 0.001). Accordingly, the LoD_95_ corresponding to a 95% detection probability of the test was determined to be 63.8 copies/µL.

However, as the assay was designed as a qualitative test, it was not intended for clinical viral load quantification. The LoD_95_ was determined using plasmid DNA under controlled conditions and may not directly reflect matrix-specific analytical sensitivity in inhibitor-rich clinical specimens.

## 4. Discussion

In this study, the inter-method agreement of a laboratory-developed qualitative CMV PCR assay was evaluated in comparison to a commercially available comparator assay using different clinical sample types, and the results were analyzed on a sample type basis. The study design and reporting process were conducted in accordance with the recommended MIQE guidelines for PCR experiments and the CLSI EP12-A2 recommendations for the evaluation of qualitative diagnostic tests [21,22,23,24]. Although the artus^®^ CMV QS-RGQ assay was used as the comparator method in this study, it is not a universally clinically validated gold standard for all non-plasma specimen types. Currently, no universally accepted clinical gold standard exists for CMV PCR detection across diverse non-plasma clinical specimen matrices. These findings primarily reflect methodological concordance between assays across different specimen matrices. Therefore, the present findings reflect analytical inter-method agreement between two molecular assays rather than definitive clinical diagnostic accuracy.

The artus^®^ CMV QS-RGQ assay amplifies a conserved 105 bp region of the CMV genome and includes a heterologous internal control to monitor extraction efficiency and PCR inhibition; however, the exact target gene region and primer/probe sequences are proprietary and not disclosed by the manufacturer. This limits the ability to perform a direct analytical comparison at the primer/probe level between the two assays.

The results obtained from BALF samples showed a moderate level of agreement between the laboratory-developed assay and the comparator assay. The latent presence of CMV in the respiratory tract and subclinical replication suggest that CMV DNA detected by PCR in BALF samples may not always reflect invasive CMV pneumonia. Indeed, Boeckh et al. emphasized that the relationship between BALF and plasma viral loads can be variable and that BALF PCR results should be interpreted carefully in the clinical context [25].

CMV PCR positivity in BAL samples alone is not sufficient for the diagnosis of CMV pneumonia and should be interpreted in conjunction with the clinical presentation, radiological findings, and, when available, histopathological data. Similarly, studies in lung transplant recipients recommend that BALF CMV PCR results be interpreted in conjunction with histopathological and clinical data [26].

In our study, the false positive and false negative results observed in BALF samples may be attributable to the biological heterogeneity of respiratory specimens and the presence of PCR inhibitors in this matrix. It has previously been demonstrated that inhibitory substances in respiratory samples can suppress amplification efficiency and contribute to variability in qualitative PCR results [17]. The comparatively lower positive percent agreement observed in BALF specimens may therefore reflect matrix-related amplification variability and heterogeneous viral distribution within the respiratory tract. These findings are consistent with previous reports indicating that CMV DNA detection in BALF does not uniformly correlate with invasive pulmonary disease and may show discordance with other specimen types [25,26]. Accordingly, the BALF results in this study should be interpreted within the analytical framework of inter-method comparison.

Although the comparator assay provides cycle threshold (Ct) values, the laboratory-developed assay evaluated in this study was designed as a qualitative test, and no quantitative comparison was performed. However, it is well recognized that discordant results between molecular assays are more likely to occur in samples with low viral loads, typically corresponding to high Ct values near the detection limit. Therefore, the discordant and weak-positive results observed in this study may be attributed to differences in analytical sensitivity and stochastic variation in samples with low viral DNA concentrations.

The high concordance observed in gastrointestinal and stool samples indicates stable inter-method agreement in these specimen types. CMV is known to be associated with gastrointestinal involvement, particularly in immunocompromised patients [27]. Previous studies have reported that CMV DNA detection in stool samples may contribute to the laboratory evaluation of intestinal CMV involvement [28].

Although stool represents a matrix enriched with potential PCR inhibitors, it has been demonstrated that appropriate nucleic acid extraction protocols and internal control strategies can substantially mitigate inhibitory effects [17,18]. In the present study, the high negative percent agreement and overall agreement observed in stool specimens support consistent analytical performance of the assay in this matrix.

The substantial inter-method agreement observed in urine samples indicates stable analytical concordance between the two assays in this specimen type. CMV urinary excretion is a well-characterized virological phenomenon, and previous studies have emphasized the analytical suitability of urine and other sterile body fluids for CMV DNA detection [28]. Urine-based PCR approaches have been widely used, particularly in the context of congenital CMV infections, where molecular detection in urine represents a well-established laboratory practice [29]. The agreement observed in the present study is consistent with previously reported analytical performance characteristics in urine specimens [28,30]. In this group, which includes colonoscopy biopsies, amniotic fluid, and intraocular fluid samples, the high level of inter-method agreement observed among the assays, as well as the stable analytical performance of the laboratory-developed qualitative CMV PCR assay in invasive and sterile body fluids, are particularly noteworthy. Previous studies have demonstrated that CMV detection in invasive specimens—including gastrointestinal tissue, amniotic fluid, and ocular fluids—may indicate direct tissue invasion, while molecular methods provide faster and more sensitive diagnostic information than histopathological approaches. However, due to the limited number of samples in this group, the findings need to be validated in larger patient series [18,28,29,31].

When all clinical specimen types were evaluated collectively, the laboratory-developed qualitative CMV PCR assay demonstrated substantial overall inter-method agreement with the comparator assay, with agreement levels classified as good to very good according to Cohen’s kappa coefficient. The observed agreement metrics across urine, stool, and invasive specimens indicate stable analytical concordance in these matrices.

The analytical sensitivity of the assay, as determined by probit regression analysis, demonstrated a LoD_95_ value of 63.8 copies/µL. While this reflects the intrinsic detection capability of the assay under controlled conditions using plasmid DNA tested without nucleic acid extraction, it should be interpreted with consideration of matrix-related effects. In non-plasma clinical specimens such as BALF and stool, the presence of PCR inhibitors and biological variability may influence detection performance. Accordingly, the analytical sensitivity findings should be interpreted in conjunction with the specimen-specific agreement results observed in this study.

In BALF samples, comparatively lower agreement and variability in positive percent agreement may reflect biological heterogeneity of respiratory specimens and matrix-related amplification variability. These findings are consistent with previous observations that CMV DNA detection in BALF does not uniformly correlate with invasive pulmonary disease and should therefore be interpreted with caution in analytical comparisons [25,26].

In the evaluation of inter-method agreement, the choice of the targeted viral genome region is also an important factor affecting test performance. In studies comparing the clinical performance of real-time PCR approaches targeting different regions of the cytomegalovirus genome, conserved gene regions such as US17 have been reported to be reliable targets. Sanghavi et al. used real-time PCR targeting the US17 and UL54 regions to assess CMV load in organ transplant recipients, and the resulting viral load results were consistent with pp65 antigenemia [32]. This study supports the reliability of the PCR target regions used for clinical monitoring. Additionally, it has been reported that in studies conducted by NIST for the development of reference material for CMV, different gene regions, including US17, were evaluated sequentially and analytically as qPCR targets [33]. In light of these findings, the preference for PCR design targeting the US17 region in our study can be considered one of the potential determinants of the overall concordance observed across different clinical sample types.

The present study has several important strengths. First, it provides a structured, specimen-type-based evaluation of inter-method agreement for a laboratory-developed qualitative CMV PCR assay across a broad spectrum of non-plasma clinical materials. To our knowledge, systematic specimen-type-based analytical evaluations of a newly developed qualitative CMV PCR assay across such a diverse range of non-plasma clinical matrices remain limited in the literature. By systematically analyzing stool, urine, bronchoalveolar lavage, colonoscopic biopsy, amniotic fluid, and intraocular fluid specimens within a unified analytical framework, the study offers a matrix-specific perspective on qualitative CMV DNA detection that is infrequently addressed in comparative PCR evaluations.

Second, the prospective and comparative design, together with adherence to internationally recognized methodological standards (CLSI EP12-A2 and MIQE guidelines), enhances the analytical rigor and reproducibility of the findings. The parallel testing of all specimens using a consistent nucleic acid extraction workflow minimizes pre-analytical variability and strengthens the internal validity of the inter-method comparison. Third, the specimen-type-based agreement analysis highlights the impact of biological and matrix-related variability on qualitative PCR performance. The observed concordance patterns across different specimen types contribute to a better understanding of how qualitative CMV detection may behave in heterogeneous clinical materials under routine laboratory conditions.

The CMV PCR assay evaluated in this study was designed as a qualitative test and was not intended for viral load quantification. Because numerical Cq/Ct values were not archived in a standardized format within the routine diagnostic workflow, quantitative comparisons were not feasible. Nevertheless, to facilitate interpretation of discordant results, a descriptive Cq category classification was applied, and detailed data are presented in Supplementary Tables S2 and S3.

Several limitations should also be acknowledged. This study was restricted to qualitative detection and did not permit quantitative assessment of viral load across specimen types. Non-plasma specimens were not paired with concurrent plasma samples, limiting evaluation of potential viral compartment relationships. Subgroup analyses—particularly for invasive specimens—were constrained by limited sample size and absence of a priori power calculation. Additionally, the single-center design may restrict generalizability. Future multicenter investigations incorporating larger cohorts and paired plasma and non-plasma analyses are warranted to further explore matrix-specific agreement patterns and extend these observations. Future studies should include multicenter validation across larger and more diverse patient populations to improve generalizability. In addition, integration of quantitative PCR approaches and standardized Ct value reporting would allow more detailed assessment of assay performance, particularly in low viral load samples and discordant cases. These approaches would further enhance the clinical applicability and robustness of the assay.

## 5. Conclusions

In conclusion, this prospective comparative analytical study demonstrates that the laboratory-developed qualitative CMV real-time PCR assay exhibits substantial inter-method agreement with a commercially available comparator assay across multiple non-plasma clinical specimen types. Agreement levels varied according to specimen matrix, reflecting inherent biological and analytical differences among sample types.

Higher concordance was observed in stool, urine, and invasive specimens, indicating stable analytical agreement in these matrices. In contrast, BALF specimens showed comparatively lower agreement, which may be attributable to heterogeneous viral distribution, lower target concentrations, and matrix-related amplification variability characteristic of respiratory samples.

The findings represent methodological concordance between two molecular assays rather than definitive clinical diagnostic accuracy. As the evaluated assay was designed as a qualitative test, it is not intended for viral load quantification, and the results reflect inter-method analytical agreement.

Overall, the data support the analytical applicability of the assay for qualitative CMV DNA detection in non-plasma specimens under routine laboratory conditions, while emphasizing specimen-specific analytical interpretation.

## Figures and Tables

**Table 1:** Distribution of clinical specimens and CMV positivity.

Specimen Type	Total Samples (*n*)	CMV Positive *n* (%)	CMV Negative *n* (%)
BALF	69	11 (15.9)	58 (84.1)
Stool	34	7 (20.6)	27 (79.4)
Urine	29	5 (17.2)	24 (82.8)
CB/AF/Ocular	34	7 (20.6)	27 (79.4)
Total	166	30 (18.1)	136 (81.9)

BALF, bronchoalveolar lavage fluid; CB, colonoscopic biopsy; AF, amniotic fluid; Ocular, intraocular fluid; CMV, cytomegalovirus. Percentages are calculated based on the total number of specimens within each specimen type.

**Table 2:** Inter-method agreement metrics by specimen type.

Specimen Type	TP	FP	FN	TN	PPA (%)	NPA (%)	OPA (%)	Cohen’s κ (95% CI)
BALF	5	7	6	51	45.5	87.9	81.2	0.36 (0.03–0.61)
Stool	6	2	1	25	85.7	92.6	91.2	0.79 (0.47–1.00)
Urine	4	1	1	23	80.0	95.8	93.1	0.74 (0.44–1.00)
CB/AF/Ocular	7	1	0	26	100	96.3	97.1	0.93 (0.75–1.00)
All specimens	22	11	8	125	73.3	91.9	88.6	0.66 (0.48–0.78)

BALF, bronchoalveolar lavage fluid; CB, colonoscopic biopsy specimens; AF, amniotic fluid; Ocular, ocular fluid; TP, true positive; FP, false positive; FN, false negative; TN, true negative; PPA, positive percent agreement; NPA, negative percent agreement; OPA, overall percent agreement; CI, confidence interval. TP, FP, FN, and TN are presented as absolute numbers.

**Table 3:** Evaluation of the analytical sensitivity (LoD_95_) of the CMV qPCR test using probit regression analysis.

Parameter	Estimate	Standard Error	z-Value	*p*-Value
Fixed (β_0_)	−5.35	0.57	−9.43	<0.001
Log_10_ (copy/µL) (β_1_)	3.88	0.38	10.20	<0.001

## Data Availability

Data supporting the findings of this study are available from the corresponding author: Murat Aral, upon reasonable request.

## Supplementary Materials

The following supporting information can be downloaded at: https://www.mdpi.com/article/10.3390/v18040417/s1: Table S1: Definition of amplification classes and their approximate correspondence to qPCR cycle threshold (Cq) ranges. Table S2: Distribution of discordant samples according to Cq category (n = 20). Table S3: In silico compatibility analysis of the CMV US17 primer and probe binding regions with reference CMV sequences.

## Abbreviations

The following abbreviations are used in this manuscript:

AF	Amniotic fluid
BALF	Bronchoalveolar lavage fluid
CB	Colonoscopic biopsy
CI	Confidence interval
CLSI	Clinical and Laboratory Standards Institute
CMV	Cytomegalovirus
κ	Cohen’s kappa coefficient
MIQE	Minimum Information for Publication of Quantitative Real-Time PCR Experiments
NIST	National Institute of Standards and Technology
NPA	Negative percent agreement
NTC	No-template control
OPA	Overall percent agreement
PCR	Polymerase chain reaction
PPA	Positive percent agreement
qPCR	Quantitative real-time polymerase chain reaction
WHO	World Health Organization
