# Supplementary material for: Inter-Method Agreement of a Laboratory-Developed Qualitative CMV PCR Assay Across Multiple Non-Plasma Clinical Specimens
Source: Viruses. 2026 Mar 27;18(4):417. doi: 10.3390/v18040417 (PMC13120338; doi:10.3390/v18040417)
Supplement: Supplementary file 1 [file viruses-18-00417-s001.zip › Supplementary Table S1.pdf]

Supplementary Table S1. Amplification class and approximate qPCR cycle correspondence

Amplification Class	Approximate qPCR Cycle (Cq)
Strong (+)	<30
Moderate (+)	30–35
Weak (+)	>35

Approximate ranges are for descriptive purposes only; since the test is qualitative, numerical Cq values are not reported.
