# Supplementary material for: Inter-Method Agreement of a Laboratory-Developed Qualitative CMV PCR Assay Across Multiple Non-Plasma Clinical Specimens
Source: Viruses. 2026 Mar 27;18(4):417. doi: 10.3390/v18040417 (PMC13120338; doi:10.3390/v18040417)
Supplement: Supplementary file 1 [file viruses-18-00417-s001.zip › Supplementary Table S2.pdf]

Supplementary Table S2. Classification of discordant samples by Cq category (n = 20)

Sample No	Sample Type	LDT	Reference Assay	Cq Category
5	Colonoscopy biopsy	Positive	Negative	Late/Weak positive (borderline)
9	Stool	Positive	Negative	Late/Weak positive (borderline)
19	Stool	Positive	Negative	Late/Weak positive (borderline)
28	Stool	Positive	Negative	Late/Weak positive (borderline)
33	BALF	Positive	Negative	Late/Weak positive (borderline)
34	BALF	Positive	Negative	Late/Weak positive (borderline)
47	BALF	Positive	Negative	Late/Weak positive (borderline)
49	Sample type unclear (excluded)	Positive	Negative	Late/Weak positive (borderline)
56	BALF	Positive	Negative	Late/Weak positive (borderline)
58	BALF	Positive	Negative	Late/Weak positive (borderline)
66	Urine	Positive	Negative	Moderate positive
79	Intraocular fluid	Positive	Negative	Late/Weak positive (borderline)
100	BALF	Positive	Negative	Late/Weak positive (borderline)
117	BALF	Positive	Negative	Late/Weak positive (borderline)
130	Urine	Positive	Negative	Moderate positive
140	Colonoscopy biopsy	Positive	Negative	Late/Weak positive (borderline)
161	BALF	Positive	Negative	Late/Weak positive (borderline)
163	BALF	Positive	Negative	Late/Weak positive (borderline)
171	BALF	Positive	Negative	Late/Weak positive (borderline)
181	Stool	Positive	Negative	Late/Weak positive (borderline)

Footnote: Cq categories represent qualitative interpretations rather than numerical Cq values, as routine testing was performed in qualitative mode. BALF = bronchoalveolar lavage fluid; LDT = laboratory-developed test. Weak positive (borderline) indicates late amplification near the limit of detection. The Cq category in this study does not represent a numerical Cq/Ct value. As the study was conducted within a routine diagnostic workflow using a qualitative testing approach, numerical Cq values were not retrospectively archived in a standardized manner. Therefore, discordant samples were reported using a descriptive classification reflecting borderline/possible low target levels, particularly for specimen types known in the literature to be associated with matrix effects and inhibition risk. Numerical Cq-based analysis was not intended.
